# Sox9-Increased miR-322-5p Facilitates BMP2-Induced Chondrogenic Differentiation by Targeting Smad7 in Mesenchymal Stem Cells

**DOI:** 10.1155/2021/9778207

**Published:** 2021-11-05

**Authors:** Yongsheng Zeng, Chengcheng Du, Pengcheng Xiao, Yiting Lei, Piao Zhao, Zhenglin Zhu, Shengqiang Gao, Bowen Chen, Shengwen Cheng, Wei Huang, Chen Zhao

**Affiliations:** ^1^Department of Orthopedic Surgery, The First Affiliated Hospital of Chongqing Medical University, No. 1 Yixueyuan Road, Yuzhong District, Chongqing 400016, China; ^2^Molecular Oncology Laboratory, Department of Orthopaedic Surgery and Rehabilitation Medicine, The University of Chicago Medical Center, Chicago, IL 60637, USA

## Abstract

Bone morphogenetic protein 2 (BMP2) induces effective chondrogenesis of mesenchymal stem cells (MSCs) by promoting Sox9 expression. However, BMP2 also induces chondrocyte hypertrophy and endochondral ossification by upregulating Smad7 expression, which leads to the disruption of chondrogenesis. In addition, Smad7 can be inhibited by Sox9. Therefore, the underlying mechanism is not clear. Currently, an increasing number of studies have shown that microRNAs play a pivotal role in chondrogenic and pathophysiological processes of cartilage. The purpose of this study was to determine which microRNA is increased by Sox9 and targets Smad7, thus assisting BMP2 in maintaining stable chondrogenesis. We found that miR-322-5p meets the requirement through next-generation sequencing (NGS) and bioinformatic analysis. The targeting relationship between miR-322-5p and Smad7 was confirmed by dual-luciferase reporter assays, qPCR, and western blotting (WB). The in vitro study indicated that overexpression of miR-322-5p significantly inhibited Smad7 expression, thus causing increased chondrogenic differentiation and decreased hypertrophic differentiation, while silencing of miR-322-5p led to the opposite results. Flow cytometry (FCM) analysis indicated that overexpression of miR-322-5p significantly decreased the rate of early apoptosis in BMP2-stimulated MSCs, while silencing of miR-322-5p increased the rate. A mouse limb explant assay revealed that the expression of miR-322-5p was negatively correlated with the length of the BMP2-stimulated hypertrophic zone of the growth plate. An in vivo study also confirmed that miR-322-5p assisted BMP2 in chondrogenic differentiation. Taken together, our results suggested that Sox9-increased miR-322-5p expression can promote BMP2-induced chondrogenesis by targeting Smad7, which can be exploited for effective tissue engineering of cartilage.

## 1. Introduction

Traumatic or degenerative cartilage defects are a challenging clinical issue, as cartilage tissue is devoid of vascular, neural, or lymphatic structures [1]. The above characteristics contribute to the poor self-healing capacity of cartilage. Thus, cartilage needs to be reestablished once injured [2]. Mesenchymal stem cells (MSCs) have been identified as ideal seed cells in cartilage tissue engineering due to their chondrogenic differentiation potential [2–4].

Bone morphogenetic protein 2 (BMP2), a member of the transforming growth factor beta (TGF-*β*) superfamily, is a potent growth factor for the induction of MSC chondrogenic differentiation [5–7]. However, BMP2 alone cannot achieve stable chondrogenesis, as it stimulates chondrogenic hypertrophic differentiation and endochondral ossification, which destroy the cartilage phenotype [3, 8, 9]. Sox9, induced significantly by BMP2, is the key transcription factor that maintains the chondrocyte phenotype and cartilage homeostasis. This molecule plays an important role in the production and protection of the extracellular matrix of articular cartilage [10–13]. A previous study by our team showed that overexpression of Sox9 enhances BMP-2-induced chondrogenic differentiation in MSCs by downregulating Smad7 expression [14–16]. However, the underlying mechanism of how Sox9 regulates Smad7 is not clear.

Increasing evidence indicates that microRNAs (miRNAs) are crucial for the regulatory network in chondrocyte differentiation and cartilage function [17, 18]. miRNAs are a class of noncoding, single-stranded, and small-molecule RNAs that are approximately 18–24 nucleotides in length. They play a crucial role in many biological processes through posttranscriptional negative regulation of target gene expression by sequence-specific binding to the 3′ untranslated regions (UTRs) of their target messenger RNAs (mRNAs) [17, 19, 20]. Therefore, we hypothesized that Sox9 could promote the expression of certain miRNAs that target and inhibit Smad7 expression.

In the present study, we investigated the function of miR-322-5p in BMP2-mediated chondrogenic and hypertrophic differentiation in MSCs. Sox9 was found to increase the expression of miR-322-5p, which targeted Smad7. Our experiments revealed that overexpression of miR-322-5p suppressed BMP2-induced MSC early apoptosis and chondrocyte hypertrophy, thus facilitating BMP2-induced chondrogenic differentiation. These findings help elucidate BMP2-mediated chondrogenic and hypertrophic differentiation, which can be exploited for BMP2-mediated cartilage tissue engineering.

## 2. Materials and Methods

### 2.1. Cell Culture and Chemicals

Mouse bone marrow MSC C3H10T1/2 and human embryonic kidney (HEK) 293 cell lines were obtained from the American Type Culture Collection (ATCC, Manassas, VA, United States). Cell lines were maintained in complete Dulbecco's modified Eagle's medium (DMEM, BioExplorer, USA) supplemented with 10% fetal bovine serum (FBS, PAN Biotech, Germany), 100 mg/ml streptomycin, and 100 U/ml penicillin at 37°C in a humidified atmosphere with 5% carbon dioxide (CO_2_). Unless mentioned otherwise, all chemicals were purchased from Thermo-Fisher Scientific or Sigma-Aldrich.

### 2.2. Construction and Generation of Recombinant Adenoviral Vectors AdBMP2, AdSox9, AdGFP, AdshSox9, and AdRFP

AdEasy technology was used to generate recombinant adenoviruses as previously described [21]. AdBMP2, AdSox9, and AdshSox9 were previously characterized [8, 14, 16, 22]; AdGFP and AdRFP were used as mock virus controls. Briefly, the full-length transcript of mouse-derived Sox9 and the coding region of human-derived BMP2 were PCR amplified and subcloned into an adenoviral shuttle vector to generate recombinant adenoviral vectors; vectors containing Sox9 or BMP2 were subsequently used to generate recombinant adenoviruses in HEK-293 cells. AdshSox9 was purchased from Vigene (Shandong, China). For monitoring infection efficiency, AdBMP2 and AdSox9 were flagged with green fluorescent protein (GFP), and AdshSox9 was labeled with red fluorescent protein (RFP).

### 2.3. Chondrogenic Differentiation of MSCs in Micromass Culture

To mimic the condensation of MSCs, we used micromass culture to induce chondrogenic differentiation as previously described [23]. C3H10T1/2 cells were infected with AdGFP, AdRFP, AdBMP2, AdSox9, or AdshSox9. To manipulate miR-322-5p expression, we infected miR-322-5P agomir and antagomir, purchased from GenePharma (Shanghai, China), by siRNA-Mate™ (GenePharma) according to the manufacturer's instructions, using agomir-NC or antagomir-NC as normal controls, respectively. Twenty-four hours after infection, the cells were collected, resuspended at a high density (∼10^5^ per 50 *μ*l of DMEM), subsequently seeded at the center of each well in 6-well plates, and then incubated in a CO_2_ incubator. Two hours after incubation, 2 ml of complete DMEM was added to each well; half of the medium was replaced every 3 days.

### 2.4. RNA Isolation and qPCR

Total RNA was isolated with RNAiso Plus (TaKaRa, China) and subjected to reverse transcription with a PrimeScript RT reagent kit (TaKaRa, China) according to the manufacturer's instructions. The qPCR experiment was performed on the CFX96 Real-Time PCR Detection System (Bio-Rad, United States) using SYBR Premix Ex Taq II kit (TaKaRa, China) under the following conditions: 95°C for 30 s, 95°C for 5 s, and 60°C for 30 s, repeating 40 cycles. GAPDH was used as the internal reference, and data were normalized by the 2^−*ΔΔ*ct^ method. The primer sequences are shown in Table 1.

### 2.5. NGS and Bioinformatic Analysis

Total RNA from both groups, BMP2+RFP and BMP2+shSox9, was extracted for NGS on the Illumina HiSeq2500 sequencer 50 SE at day 6 after adenoviral infection. Then, data were analyzed to generate volcano plots, Venn diagrams, and scatter plots at https://www.omicstudio.cn/tool [24]. Prediction of miRNAs targeting Smad7 was performed in the databases miRanda, StarBase, and TargetScan.

### 2.6. Dual-Luciferase Reporter Assay

For detection of the interaction between Smad7 and miR-322-5p, plasmids containing mutant-type Smad7-3′UTR (Smad7-3′UTR-MT) and wild-type Smad7-3′UTR (Smad7-3′UTR-WT) were constructed. When HEK-293 cells cultured in 24-well plates reached 80% confluency, 50 nM miR-322-5p agomir or NC was cotransfected into cells with 2 *μ*g plasmids mediated by Lipofectamine 2000 (Invitrogen). Moreover, Renilla luciferase- (RL-) loaded pRL-TK was transfected as an internal control. After 48 h, the Dual-Luciferase Reporter Gene Assay Kit (Beyotime) was used to detect the intensity of RL and firefly luciferase (FL) according to the manufacturer's protocol. Consequently, the ratio of FL to RL reflected the suppressive effect of miR-322-5p on Smad7. Each group had five duplicate wells.

### 2.7. Western Blotting (WB)

Protein extraction was performed with lysis buffer, radioimmunoprecipitation assay (RIPA) buffer (Beyotime Biotechnology, China) containing 1% phenylmethanesulfonyl fluoride (PMSF) (Beyotime Biotechnology, China), and subsequent sonication. After centrifugation, the supernatant was boiled for denaturation and determination of total protein concentration using the BCA protein assay kit (Beyotime Biotechnology, China). Equivalent amounts of protein were loaded for electrophoresis on 7-10% SDS-PAGE gels (Omni-Easy™ One-Step PAGE Gel Fast Preparation Kit, EpiZyme, China) and transferred to polyvinylidene fluoride membranes (PVDF, 0.2 *μ*m, Bio-Rad). After the membranes were blocked with 5% skim milk at room temperature for 1 h, proteins were incubated overnight at 4°C with the following primary antibodies: Sox9 (Zen Bio, 1 : 2000), COL2A1 (Abcam, 1 : 3000), COL10A1 (Santa Cruz Biotechnology; 1 : 1000), Smad7 (Santa Cruz,1 : 1000), and GAPDH (Zen Bio, 1 : 2000). After the membranes were washed with TBST, they were incubated with the corresponding secondary antibodies (goat anti-rabbit IgG, 1 : 10000, Zen Bio) for 1 h at room temperature. Following sequential washing with TBST and TBS, the target proteins were detected by an ECL detection kit (Thermo Fisher Scientific), and ImageJ software was used for quantification of band density.

### 2.8. Apoptosis Detection by Flow Cytometry

Cells were treated with trypsin (0.25%) and monitored under a microscope throughout the process of digestion, which was immediately stopped by adding DMEM containing FBS when most of the cells became round. After centrifugation, the cells were repeatedly resuspended in PBS gently and centrifuged twice, followed by resuspension in 500 *μ*l of PBS for immediate detection on CytoFLEX.

### 2.9. Mouse Fetal Limb Explant Culture

Forelimbs of mouse embryos (E18.5) were dissected of skin and most soft tissue, except periosteum, under sterile conditions and incubated in DMEM containing 0.5% bovine serum albumin (BSA, Sigma), 50 *μ*g/ml ascorbic acid, 1 mM *β*-glycerophosphate, 100 mg/ml streptomycin, and 100 U/ml penicillin at 37°C in a humidified atmosphere with 5% carbon dioxide (CO_2_) for up to 14 days as previously described [25, 26]. Five samples were cultured in each well. Upon the initiation of incubation, the skin-free limbs were infected by adding AdBMP2, AdSox9 or AdshSox9, miR-322-5P agomir, or antagomir to the culture medium. Half of the medium was changed every second day. For monitoring the survival of the cells in the forelimbs, GFP and RFP signals were observed under a microscope. The tissues were fixed for histological evaluation after 14 days of culturing.

### 2.10. Subcutaneous MSC Implantation

The animal use and care and experimental procedures were approved by the Chongqing Medical University Animal Care and Use Committee. The subcutaneous stem cell implantation procedure was carried out as described [3, 14]. Briefly, C3H10T1/2 cells were infected with AdBMP2, AdGFP, AdRFP, AdSox9, AdshSox9, miR-322-5P agomir, and antagomir. Twenty-four hours after transfection, the cells were collected and resuspended in PBS-diluted Matrigel (Corning) for subcutaneous injection into the flanks of athymic nude mice (4 weeks old, female, *n* = 3/group, 4∗10^6^ cells per injection). Four weeks after injection, the animals were sacrificed for collection of ectopic masses. Following fixation in 4% paraformaldehyde (Servicebio, Wuhan, China) for 24 h at room temperature, the masses were subjected to ethylenediaminetetraacetic acid (EDTA) for decalcification at 4°C for 14 days, followed by embedding in paraffin. Serial 5 *μ*m thick sections were processed for special staining and histological evaluations.

### 2.11. Histological Evaluation: Hematoxylin and Eosin (H&E), Alcian Blue Staining, and Masson's Trichrome

After sequential deparaffinization with xylene and rehydration with graded ethanol, H&E, Masson's trichrome, and Alcian Blue staining was performed using a standard protocol as described previously [8, 14, 16]. Briefly, the deparaffinized samples were first subjected to antigen retrieval and fixation, followed by H&E and Masson's trichrome staining. A light microscope (Olympus, Japan) was used for histological evaluation.

### 2.12. Immunohistochemistry Assay

After sequential deparaffinization with xylene and rehydration with graded ethanol, sections were boiled in 10 mM citrate buffer at 95–100°C for 10 min for antigen retrieval, rinsed in 3% H_2_O_2_ at room temperature for 10 min to inhibit endogenous peroxidase activity, and blocked with 10% goat serum at room temperature for 10 min. Then, the sections were incubated with primary antibodies against collagen 2*α*1 (COL2A1) (Abcam, 1 : 400) and collagen 10*α*1 (COL10A1) (Abcam, 1 : 200) at 4°C overnight. After washing, the sections were incubated with secondary antibody at 37°C for 30 min, followed by incubation with streptavidin–HRP conjugate for 20 min at room temperature. Staining without primary antibody was used as a negative control. A microscope (Olympus, Japan) was used for imaging.

### 2.13. Statistical Analysis

All experiments were performed at least three times independently. Data are expressed as the mean ± standard deviation (SD) and were analyzed with SPSS software (Version 21, IBM). Statistical analyses were conducted using one-way analysis of variance and Student's *t*-test; *p* < 0.05 was considered statistically significant.

## 3. Results

### 3.1. Silencing Sox9 Inhibited BMP2-Induced Chondrogenesis

**Figure 1:**
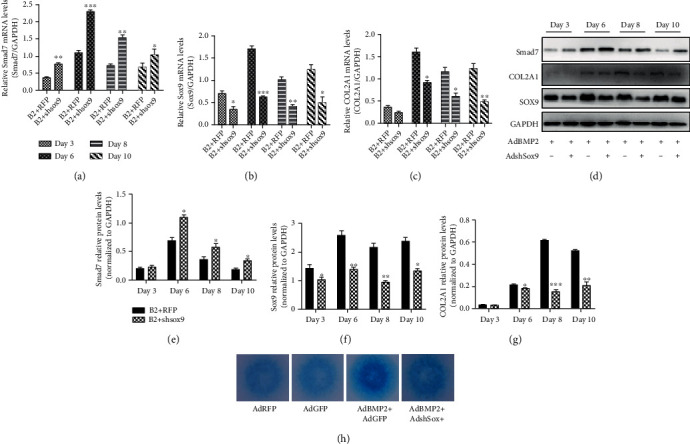
Silencing Sox9 inhibited BMP2-induced chondrogenesis. (a–c) Gene expression detected by qPCR revealed that silencing Sox9 inhibited BMP2-induced expression of Sox9 and COL2A1, while it promoted that of Smad7. (d–g) WB mimicked the results of qPCR. (h) Alcian Blue staining of micromasses demonstrated weakened chondrogenic capacity of shSox9-treated MSCs. ^∗^*p* < 0.05, ^∗∗^*p* < 0.01, and ^∗∗∗^*p* < 0.001, comparison with group AdBMP2 at corresponding time points. B2: BMP2.

AdBMP2 was infected in C3H10T1/2 cells with or without AdshSox9. Gene expression detected by qPCR at days 3, 6, 8, and 10 after infection revealed that silencing Sox9 inhibited BMP2-induced Sox9 and COL2A1 expression while promoting BMP2-induced Smad7 expression (Figures 1(a)–1(c)). The WB results were consistent with the results of qPCR (Figures 1(d)–1(g)). Micromasses infected with AdRFP, AdGFP, AdBMP2, and AdBMP2+AdshSox9 were cultured for 7 days before being subjected to Alcian Blue staining, which demonstrated weakened staining of the AdBMP2+AdshSox9 group compared with the AdBMP2+AdRFP group. These results indicated that silencing Sox9 inhibited BMP2-induced chondrogenesis.

### 3.2. NGS and Bioinformatic Analyses Demonstrated That Silencing of Sox9 Decreased the Expression of miR-322-5p, Which Was Predicted to Target Smad7

**Figure 2:**
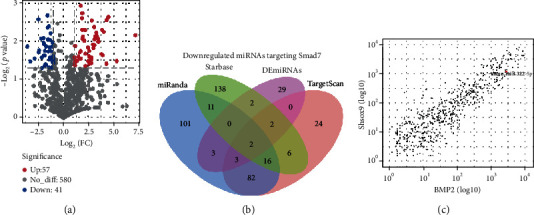
The NGS and bioinformatic analysis demonstrated that silencing of Sox9 decreased the expression of miR-322-5p which was predicted to target Smad7. (a) The volcano plot showed the upregulated and downregulated miRNAs in the BMP2+shSox9 group. (b) The Venn diagram analysis revealed that only two downregulated miRNAs, including miR-322-5p, appeared in all three databases. (c) The correlation coefficient between groups was 0.9, and miR-322-5p was marked. FC: fold change.

The volcano plot (Figure 2(a)) showed that 41 and 57 miRNAs had downregulated and upregulated expression, respectively, in the BMP2+shSox9 group. After prediction for miRNAs targeting Smad7 in the databases miRanda, StarBase, and TargetScan, Venn diagram analysis revealed that only two miRNAs among the miRNAs with downregulated expression in the BMP2+shSox9 group appeared in all three databases (Figure 2(b)). Consequently, miR-322-5p, but not miR-181-5p, was the focus of the following experiments. Next, the scatter plot showed that the correlation coefficient was 0.9, which suggested a reliable trend between the groups (Figure 2(c)).

### 3.3. The Sox9/miR-322-5p/Smad7 Axis Was Confirmed by qPCR, WB, and Dual-Luciferase Reporter Assays

**Figure 3:**
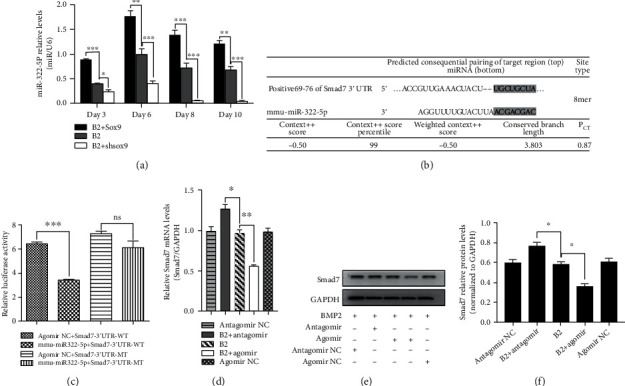
The Sox9/miR-322-5p/Smad7 axis was confirmed by qPCR, WB, and dual-luciferase reporter assay. (a) Relative expression levels of miR-322-5p among groups at different time points. (b) Predicted consequential pairing of Smad7 and miR-322-5p. (c) Dual-luciferase reporter assay confirmed the targeting relationship between Smad7 and miR-322-5p. (d) qPCR revealed the silencing effect of miR-322-5p on Smad7. (e, f) Results of WB were consistent with those of qPCR. Agomir represents mimics of miR-322-5p; antagomir represents blocker of miR-322-5p. ^∗^*p* < 0.05, ^∗∗^*p* < 0.01, and ^∗∗∗^*p* < 0.0001. NC: negative control; B2: BMP2.

Total RNA extraction at days 3, 6, 8, and 10 postinfection was performed on C3H10T1/2 cells infected with AdBMP2+AdSox9, AdBMP2, and AdBMP2+AdshSox9. Subsequently, the expression of miR-322-5p was detected by qPCR, which suggested an increased response by overexpressing Sox9 and a downregulated response by silencing Sox9 (Figure 3(a)). According to the prediction on TargetScan, miR-322-5p matched the position 69-76 of the Smad7 3′UTR (Figure 3(b)) [27]. To verify the targeting relationship between miR-322-5p and Smad7, we used antagomirs and agomirs to promote and inhibit miR-322-5p functions, respectively. The sequences are shown in Table 2. Given the expression of BMP2, WB results, in accordance with the qPCR results, showed decreased expression of Smad7 upon miR-322-5p agomir transfection and increased expression of Smad7 upon miR-322-5p antagomir transfection (Figures 3(c)–3(e)). Furthermore, a dual-luciferase reporter assay was performed and demonstrated significantly lower luciferase activity in the mmu-miR-322-5p+Samd7-3′UTR-WT group than in the agomir NC+Smad7-3′UTR-WT group; moreover, no significant difference was observed between the mmu-miR-322-5p+Samd7-3′UTR-MT and agomir NC+Smad7-3′UTR-MT groups (Figure 3(f)).

### 3.4. Smad7-Induced Early Apoptosis Was Negatively Correlated with the Expression Level of miR-322-5p

**Figure 4:**
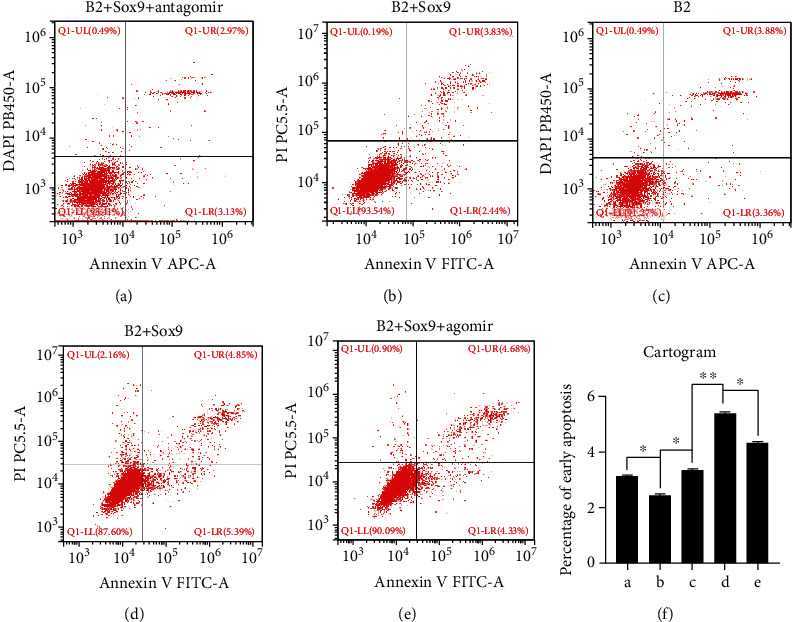
Flow cytometry was performed for detection of early apoptosis. (a–e) The percentage of early apoptosis in each group was shown. (f) ^∗^*p* < 0.05 and ^∗∗^*p* < 0.01. B2: BMP2.

To determine the effect of miR-322-5p on Smad7-related early apoptosis, we performed flow cytometry (FCM). As indicated by the results, overexpression of Sox9 obviously decreased the early apoptosis rate (Figures 4(b) and 4(c)), which was partly restored by forced expression of miR-322-5p antagomir (Figures 4(a) and 4(b)); moreover, silencing of Sox9 remarkably upregulated the early apoptosis rate (Figures 4(c) and 4(d)), which was partially reversed by use of the miR-322-5p agomir (Figures 4(d) and 4(e)). The cartogram shows the early apoptosis rate in each group (Figure 4(f)).

### 3.5. BMP2-Induced Chondrocyte Hypertrophy in Fetal Mouse Forelimb Explants Was Inhibited by miR-322-5p

**Figure 5:**
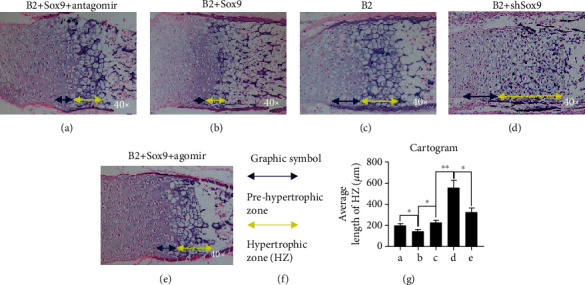
BMP2-induced chondrocyte hypertrophy in fetal mouse forelimb explant was inhibited by miR-322-5p. (a–e) Forelimbs were subjected to H&E staining for histological evaluation. (f) The arrows in dark blue and yellow represent the length of prehypertrophic and hypertrophic zone (HZ), respectively. (g) ^∗^*p* < 0.05; ^∗∗^*p* < 0.01. B2: BMP2.

After culture for 14 days, fetal mouse forelimbs were subjected to sectioning and H&E staining to evaluate the length of the hypertrophic zone. The results demonstrated that overexpressing Sox9 reduced the BMP2-induced hypertrophic zone, which was partly reversed by forced expression of the miR-322-5p antagomir (Figures 5(a)–5(c)); moreover, silencing of Sox9 extended the hypertrophic zone, which was partly reversed by the use of the miR-322-5p agomir (Figures 5(c)–5(e)). The arrows in dark blue and yellow represent the lengths of the prehypertrophic and hypertrophic zones (HZs), respectively (Figure 5(f)). The cartogram shows the length of the HZ in each group (Figure 5(g)).

### 3.6. BMP2-Induced Chondrogenesis Was Enhanced by miR-322-5p In Vitro

**Figure 6:**
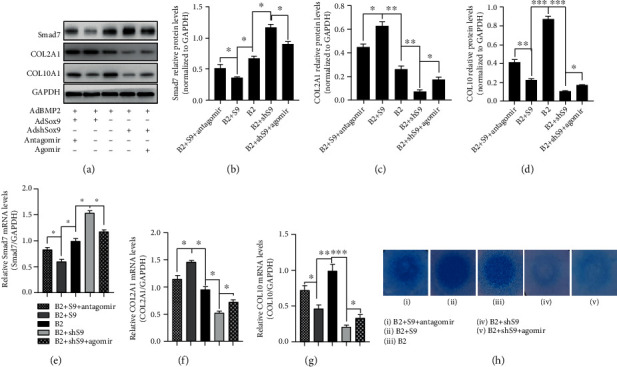
BMP2-induced chondrogenesis was enhanced by miR-322-5p. (a–d) WB revealed that overexpression of miR-322-5p inhibited expression of Smad7 and COL10A1 and promoted that of COL2A1; silencing of miR-322-5p brought the opposite changes. (e–g) Results of qPCR mimicked those of WB. (h) Alcian Blue staining supported the effect of miR-322-5p in facilitating BMP2-induced chondrogenesis. ^∗^*p* < 0.05, ^∗∗^*p* < 0.01, and ^∗∗∗^*p* < 0.0001. B2: BMP2; S9: Sox9; shS9: shSox9.

C3H10T1/2 cells infected with AdBMP2+AdSox9+antagomir, AdBMP2+AdSox9, AdBMP2, AdBMP2+AdshSox9, or AdBMP2+AdshSox9+agomir were cultured in micromasses for 7 days, followed by total protein extraction and Alcian Blue staining. As shown by the blots, overexpressing Sox9 promoted BMP2-induced expression of COL2A1 but inhibited that of COL10A1 and Smad7 (Figures 6(a)–6(d)). When Sox9-induced miR-322-5p expression was silenced by antagomir, the expression of all the markers above was partly reversed (Figures 6(a)–6(d)). However, silencing Sox9 inhibited BMP2-induced expression of COL2A1 but promoted that of COL10A1 and Smad7 (Figures 6(a)–6(d)). When miR-322-5p expression was induced by using agomir, the results were also partly reversed (Figures 6(a)–6(d)). In addition, expression at the transcriptional level supported the results of WB analysis (Figures 6(e)–6(g)). Furthermore, Alcian Blue staining suggested that the Sox9-enhanced staining was weakened by the antagomir (Figure 6(h) i–iii); moreover, the shSox9-weakened staining was enhanced by the use of the miR-322-5p agomir (Figure 6(h) iii–v).

### 3.7. BMP2-Induced Chondrogenesis Was Enhanced by miR-322-5p In Vivo

**Figure 7:**
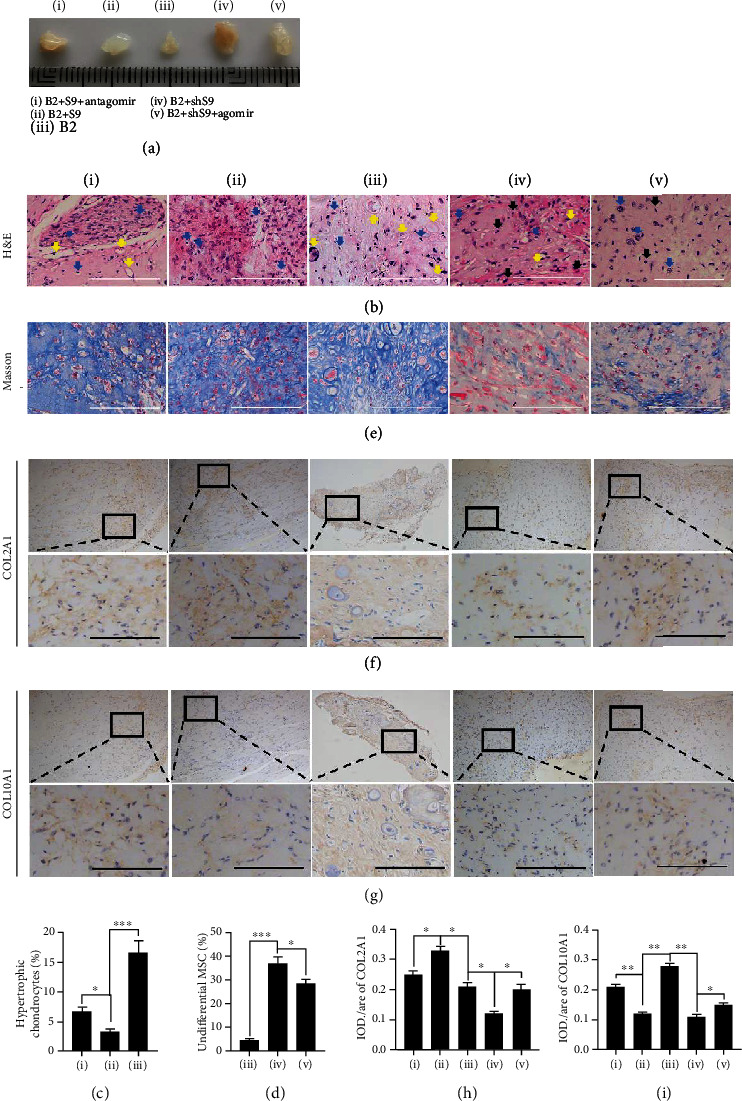
BMP2-induced chondrogenesis was enhanced, and chondrocyte hypertrophy was weakened by miR-322-5p in vivo. (a) Masses were retrieved after 4 weeks of injection. (b) Overexpressing Sox9 decreased the number of hypertrophic chondrocytes (yellow arrow), which was partly reversed by the use of the miR-322-5p antagomir ((b) i–iii); moreover, silencing Sox9 decreased the number of differentiated chondrocytes (blue arrow) and increased the number of undifferentiated MSCs (dark arrow), which was partly reversed by forced expression of the miR-322-5p agomir ((b) iii–v). (c, d) Quantitative analysis of randomized three fields in each group. (e) According to the Masson staining, increased formation of cartilage tissue promoted by Sox9 was partly reversed by adding of miR-322-5p antagomir, while decreased production of cartilage tissue caused by silencing Sox9 was partly reversed by forced expression of miR-322-5p agomir. (f, g) IHC showed that overexpression of Sox9 increased the synthesis of COL2A1 and decreased the generation of COL10A1, and both were partly reversed by use of miR-322-5p antagomir; simultaneously, silence of Sox9 decreased the production of COL2A1 and increased the formation of COL10A1, and both were partly reversed by use of miR-322-5p agomir. (h, i) Quantitative analysis of positive-stained area. Integral optical density/area (IOD/area) was calculated with Image Pro Plus software. Scale bar = 150 *μ*m; ^∗^*p* < 0.05, ^∗∗^*p* < 0.01, and ^∗∗∗^*p* < 0.001.

For determination of whether miR-322-5p is effective in silencing Smad7, thus facilitating BMP2-induced chondrogenesis in vivo, C3H10T1/2 cells infected with AdBMP2+AdSox9+antagomir, AdBMP2+AdSox9, AdBMP2, AdBMP2+AdshSox9, AdBMP2+AdshSox9+agomir, AdGFP, AdRFP, AdSox9, AdSmad7, miR-322-5P agomir, and antagomir were subcutaneously injected into the flanks of nude mice. The results indicated that no detectable masses were formed in the cells infected with AdGFP, AdRFP, AdSox9, miR-322-5P agomir, or antagomir alone. Masses were retrieved after 4 weeks of injection. The amount of Sox9 and miR-322-5p was positively correlated with hyaline cartilage-like appearance (Figure 7(a)).

Based on the histological evaluation, overexpressing Sox9 decreased the number of hypertrophic chondrocytes (yellow arrow), which was partly reversed by the use of the miR-322-5p antagomir (Figures 7(b) i–iii and 7(c)); moreover, silencing Sox9 decreased the number of differentiated chondrocytes (blue arrow) and increased the number of undifferentiated MSCs (dark arrow), which was partly reversed by forced expression of the miR-322-5p agomir (Figures 7(b) iii–v and 7(d)). According to Masson staining, the increased formation of cartilage tissue promoted by Sox9 was partly reversed by the addition of the miR-322-5p antagomir (Figure 7(e) i–iii), while the decreased production of cartilage tissue caused by silencing Sox9 was partly reversed by forced expression of the miR-322-5p agomir (Figure 7(e) iii–v).

Furthermore, immunohistochemistry showed that overexpression of Sox9 increased the synthesis of COL2A1 and decreased the generation of COL10A1, and both changes were partly reversed by use of the miR-322-5p antagomir (Figures 7(f), 7(g) i–iii, 7(h), and 7(i)); moreover, silencing of Sox9 decreased the production of COL2A1 and increased the formation of COL10A1, and both changes were partly reversed by use of miR-322-5p agomir (Figures 7(f), 7(g) iii–v, 7(h), and 7(i)).

## 4. Discussion

Articular cartilage defects caused by trauma or degeneration are increasing yearly with the development of society and changes in people's living habits. However, the repair of defective cartilage is still a challenging issue worldwide. BMP2-induced chondrogenesis of MSCs has been widely accepted as a chondrogenic model in tissue engineering. However, BMP2 induces not only chondrogenesis but also endochondral ossification [8, 14]. Previous studies by our team have shown that overexpression of Sox9 promotes BMP2-induced chondrogenesis and may function through the suppressive effect of Sox9 on Smad7 [14, 16]. The current study clarified that silencing Sox9 weakened BMP2-induced chondrogenesis by upregulating Smad7 expression. The mechanism may be that Sox9 induces miR-322-5p, which binds to the 3′UTR of Smad7 and inhibits its function.

Sox9 is highly increased by BMP2, and it facilitates BMP2-induced chondrogenic differentiation [11, 16, 28]. This molecule directly regulates the production of COL2A1, which is chondrocyte specific [29, 30]. Smad7 is an inhibitor in the Smad family; it plays an inhibitory role in the TGF*β*/BMP pathway, which is crucial in the processes of chondrogenesis [31–33]. Previous studies have found that BMP2-induced high expression of Smad7 serves as the key inhibitor of chondrogenic differentiation [32, 34]. In addition, the hypertrophic differentiation of chondrocytes, marked with COL10A1 [15, 35], was caused by Smad7, at least in part, during chondrogenic differentiation [15]. Further research revealed that Smad7 inhibited the formation of cartilaginous tissue induced by BMP2 by suppressing the P38 and Smad1/5/8 pathways [32, 36].

Increasing research has revealed the important roles of miRNAs in the processes of chondrocyte formation and the pathophysiological function of cartilage. In this research, NGS and bioinformatic analysis found that miR-322-5p expression was downregulated by silencing Sox9, and the results were further clarified by qPCR. In addition, overexpression of Sox9 upregulated miR-322-5p expression. These results confirmed that Sox9 upregulates miR-322-5p expression. Moreover, the dual-luciferase reporter assay confirmed the targeting relationship between miR-322-5p and Smad7. Subsequently, in vitro experiments also showed that miR-322-5p could indeed inhibit the expression of Smad7 at the transcriptional and translational levels.

Currently, there are only a few reports on the biological functions of miR-322-5p. A previous report showed that miR-322-5p was involved in cardiac hypertrophy in rats with pulmonary hypertension by targeting IGF-1 [37]. In addition, miR-322-5p is involved in FAM3B-mediated hyperglycemic vascular smooth muscle proliferation and migration [38]. RNA sequencing based on cartilage-derived progenitor and stem cells identified miR-322-5p as one of the core regulatory molecules during the progression of OA [39]. In another RNA sequencing analysis performed by our team, high expression of miR-322-5p was positively correlated with BMP-2-induced chondrogenesis (data not shown). It was reported that overexpression of miR-322-5p activated the TGF-*β* pathway, which is important in maintaining the homeostasis of articular cartilage [37, 38, 40]. Given that previous reports and data have shown the correlation of miR-322-5p with cartilage homeostasis and chondrogenesis, this molecule was predicted to be more associated with Smad7 [41]. Thus, when the final two miRNAs were screened out, we focused on miR-322-5p for subsequent experiments. To the best of our knowledge, this is the first study to reveal the regulatory effect of miR-322-5p on Smad7 and chondrogenesis.

## 5. Conclusions

In conclusion, our findings suggested that the Sox9/miR-322-5p/Smad7 regulatory axis exists during BMP2-induced chondrogenesis and that Sox9-increased miR-322-5p can target Smad7, thus inhibiting BMP2-induced chondrocyte hypertrophy and assisting in maintaining stable chondrogenesis.

## Figures and Tables

**Table 1:** The primer sequences used for qRT-PCR.

Genes	Primer sequences (forward: 5′-3′)	Primer sequences (reverse: 5′-3′)
Smad7	AAGATCGGCTGTGGCATC	CCAACAGCGTCCTGGAGT
COL2A1	CAACACAATCCATTGCGAAC	TCTGCCCAGTTCAGGTCTCT
Sox9	AGCTCACCAGACCCTGAGAA	TCCCAGCAATCGTTACCTTC
COL10A1	TGCTGCCCTGGTCTTACTCT	GCCTTGGGATCCTAAACCT
GAPDH	CTACACTGAGGACCAGGTTGTCT	TTGTCATACCAGGAAATGAGCTT

**Table 2:** The sequences used for miR-322-5p function regulation.

Genes	Primer sequences (5′ to 3′)
mmu-miR-322-5p agomir	CAGCAGCAAUUCAUGUUUUGGA CAAAACAUGAAUUGCUGCUCUU
mmu-miR-322-5p antagomir	UCCAAAACAUGAAUUGCUGCUG

## Data Availability

Data will be available on request.
